# Selective Interaction of Heparin with the Variable Region 3 within Surface Glycoprotein of Laboratory-Adapted Feline Immunodeficiency Virus

**DOI:** 10.1371/journal.pone.0115252

**Published:** 2014-12-18

**Authors:** Qiong-Ying Hu, Elizabeth Fink, Chris K. Grant, John H. Elder

**Affiliations:** 1 School of Medicine, Taizhou University, Taizhou, Zhejiang, China; 2 Department of Immunology and Microbial Science, The Scripps Research Institute, La Jolla, California, United States; 3 Custom Monoclonals International, Inc., W. Sacramento, California, United States; German Primate Center, Germany

## Abstract

Heparan sulfate proteoglycans (HSPG) can act as binding receptors for certain laboratory-adapted (TCA) strains of feline immunodeficiency virus (FIV) and human immunodeficiency virus (HIV). Heparin, a soluble heparin sulfate (HS), can inhibit TCA HIV and FIV entry mediated by HSPG interaction in vitro. In the present study, we further determined the selective interaction of heparin with the V3 loop of TCA of FIV. Our current results indicate that heparin selectively inhibits infection by TCA strains, but not for field isolates (FS). Heparin also specifically interferes with TCA surface glycoprotein (SU) binding to CXCR4, by interactions with HSPG binding sites on the V3 loop of the FIV envelope protein. Peptides representing either the N- or C-terminal side of the V3 loop and containing HSPG binding sites were able to compete away the heparin block of TCA SU binding to CXCR4. Heparin does not interfere with the interaction of SU with anti-V3 antibodies that target the CXCR4 binding region or with the interaction between FS FIV and anti-V3 antibodies since FS SU has no HSPG binding sites within the HSPG binding region. Our data show that heparin blocks TCA FIV infection or entry not only through its competition of HSPG on the cell surface interaction with SU, but also by its interference with CXCR4 binding to SU. These studies aid in the design and development of heparin derivatives or analogues that can inhibit steps in virus infection and are informative regarding the HSPG/SU interaction.

## Introduction

Heparan sulfate proteoglycans (HSPG) are a type of glycosaminoglycans (GAG) that participate in a number of biological processes as diverse as cell adhesion and migration [1]–[3], cell growth and proliferation [4], [5], inflammation [2], [6], angiogenesis [7], [8], tumor metastasis [5], [9], [10], or cellular attachment of many viruses [11]–[14], including retrovirus family members such as HIV and FIV [15]–[18]. The diverse biological functions of HSPG are commonly mediated by HS–protein binding. However, there have been relatively few studies of HS–protein binding at the molecular level [19]. As highly sulfated heparin-like IdoA-(1→4)-GlcNS disaccharide (NS) domains are the functionally significant parts of HS in HS–protein binding [20], [21], more abundant heparin and heparin-derived oligosaccharides have been used as models for HS.

Heparin is biosynthesized as heparin proteoglycan, which consists of a unique core protein (serglycin) and multiple heparin polysaccharide chains [22], with more and longer polysaccharide chains than HSPG. Heparin is well known for its anti-coagulant activity and has been used clinically as an anti-coagulant for over 70 years [23]. Other biological activities include release of lipoprotein lipase and hepatic lipase [24], inhibition of complement activation [25], inhibition of angiogenesis [26], [27], modulation of tumor growth and metastasis [27]–[29] and antiviral activity [30]–[34]. Because heparin has therapy potential for function as a tumor metastasis modulator [27], [29], antiviral interference [30]–[34], and also serves as a model for the interaction of proteins with cell-surface HSPG [21] described above, it is of great significance to further study and understand heparin/protein interaction.

Our previous studies showed that Laboratory-adapted strains (TCA) of FIV can bind to HSPG through a HSPG binding region that involves both the N-terminal and the C-terminal sides of the V3 loop, thus facilitating productive infection of adherent cell lines (HSPG^++^, CXCR4^+^, CD134^-^) such as CrFK and G355-5 that lack expression of the normal primary binding receptor, CD134 [35].

In the present study, we further explored the treatment potential of heparin for FIV infection in order to characterize the molecular mechanism of action. Our current results indicate that heparin blocks TCA FIV infection or entry not only by competition of HSPG on the cell surface interaction with SU, but also by interference with CXCR4 binding to SU.

## Results

### Selective inhibition of TCA FIV productive infectivity by heparin

It has been reported that heparin and dextran sulfate can inhibit HIV viral infectivity [32], [36]. To investigate the anti-viral activity of heparin on FIV, we tested the effect of heparin on the infection by FS and TCA FIV. PPRcr is a variant of FIV-PPR with broad host range [35] and our previous studies [35] show that PPRcr can productively infect CrFK and G355-5 cells (HSPG^++^, CXCR4^+^, CD134^-^). At the same time, PPRcr maintains the ability to infect Gfox cells (CrFK cells engineered to over-express CD134, CXCR4^+^, HSPG^+^) [35].

**Figure 1:**
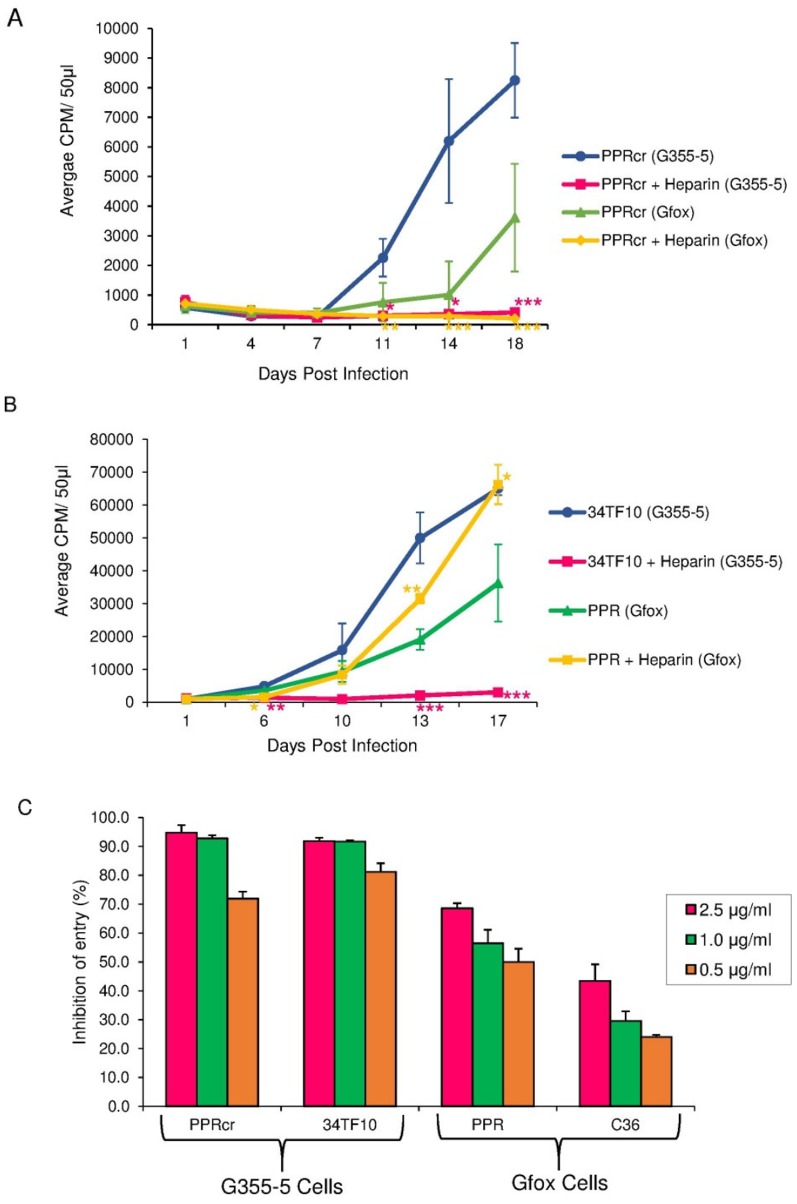
Selective inhibition of TCA FIV infectivity by heparin. (A) Productive infection assay of FIV-PPRcr inhibited by heparin in G355-5 and Gfox cells. (B) Productive infection assay of FIV-34TF10 in G355-5 and FIV-PPR in Gfox cells inhibited by heparin. Virus growth in cells was evaluated as CPM by a reverse transcriptase activity assay over time. Heparin was used at 20 µg/ml. Results are means and standard deviations (SD) for three independent determinations. ***p<0.001; **p<0.01; *p<0.05; as compared to the untreated control group, (C). Effect of heparin on FIV TCA entering G355-5 cells and FS entering Gfox cells. Entry assay were performed in the presence or absence of heparin at indicated concentrations. Values are inhibition percentage calculated as described in “Materials and Methods”. Results are means and standard deviations (SD) for three independent determinations. ***p<0.001; **p<0.01; *p<0.05; as compared to the FIV TCA groups.

Our results (Fig. 1A) showed that heparin can effectively inhibit the productive infection of PPRcr in G355-5 cells, with a significantly lower CPM values were detected between day 11 (*p*<0.05) and 18 (*p*<0.001) post-infection. Similar observation was seen in Gfox cells. CPM values in the heparin-treated group in Gfox cells were markedly lower than those of untreated control from day 11 (*p*<0.01) to day 18 (*p*<0.001) after infection. Similar to some other TCA FIV [50], the infection of PPRcr is mainly recorded as massive syncitia in the target cells with detection of viral antigen by staining. As the target cells for PPRcr produce very little virus and it takes a long time to record an increase in CPM values by RT assay, we performed the infection assays for more than two weeks and used viruses with CPM>100 K, to make sure the viruses have infectivity in the target cells.

To see whether heparin can also block the productive infections caused by other FIV isolates, we determined the effect of heparin on 34TF10 (TCA) in G355-5 cells and PPR (FS) in Gfox cells. Equivalent infectious doses of each virus were used. We found that heparin can completely interfere with the productive infection of 34TF10 in G355-5 cells (Fig. 1B), with remarkably lower CPM values detected between day 6 (*p*<0.01) and 17 (*p*<0.001) post-infection. In contrast, the effect of heparin on PPR infection in Gfox cells showed a different pattern (Fig. 1B). On day 6 post-infection of PPR in Gfox cells, CPM values in the heparin-treated group were lower than those of untreated control (*p*<0.05); but on day 13 post-infection, CPM values in the heparin-treated group were significantly higher than those of the untreated control (*p*<0.01, Fig. 1B). A similar phenomenon was observed on day 17 post-infection (*p*<0.05, Fig. 1B), indicating that heparin did not inhibit the productive infection of PPR in Gfox cells. Likewise, heparin did not block infection of Gfox cells by FS isolate, FIV C36 (data not shown). Our findings indicate that heparin can selectively inhibit the productive infection of FIV TCA, but not FIV FS, which is consistent with the report that polyanions (such as heparin and dextran sulfate) selectively inhibit HIV infection of CXCR4^+^ or CCR5^+^/CXCR4^+^ cells, but not CXCR4^-^/CCR5^+^ cells [36].

To determine whether the effect of heparin on viral infectivity is involved in the steps of virus binding and entry, a single-round infection assay (entry assay) was performed. The results (Fig. 1C) showed that heparin greatly inhibited the entry of FIV TCA PPRcr and 34TF10 into permissive G355-5 cells. Interestingly, some inhibition was also observed for entry by FS FIV-PPR and FIV-C36 in Gfox cells, but to a lesser degree than observed for TCA strains in G355-5 cells. Heparin at a high concentration (2.5 µg/ml), blocked PPRcr and 34TF10 entry (Fig. 1C, left two panels) by >90%; PPR and C36 entry (Fig. 1C, right two panels) was inhibited by <70% (*p*<0.01). At a low concentration of 0.5 µg/ml, heparin could still block PPRcr or 34TF10 entry (Fig. 1C, left two panels) with the inhibition ratio>70%, but inhibited PPR or C36 entry (Fig. 1C, right two panels) with by less than 50% (*p*<0.01) Thus, TCA FIV appears to be more susceptible to heparin inhibition than is FS FIV. The modest effect of heparin on FS FIV entry may explain the inhibition of productive infectivity by heparin at the early stage of infection (<10 days). However, when FS was overridden at the late stage of infection (>10 days) with high titer, heparin no longer has an effective action (Fig. 1B).

### Heparin inhibits TCA but not FS FIV SU binding to CXCR4

Although soluble dimeric FIV SU-Fc does not fully represent the behavior of the functional form of the SU, it is still a useful tool for analyzing a variety of interactions between SU and receptors [16], [35]–[37]. To determine whether the inhibition of viral infectivity by heparin correlated with the ability of heparin to selectively interfere with FIV surface glycoprotein (SU) interaction with receptors, we employed FACS analyses to assess the influence on receptor binding to CD134, HSPG and CXCR4 using specific target cells bearing each receptor. As previously reported [38], heparin can inhibit FIV TCA SU-Fc binding to CrFK cells but cannot inhibit FIV FS SU-Fc binding to Gfox cells. Which suggests that FIV TCA display significantly more sensitivity to heparin inhibition than does FS, at least partially due to its complete interference with TCA SU binding to HSPG and its weak or undetectable interference with FS SU binding to CD134 by heparin.

Since both TCA and FS FIV use receptor CXCR4 for entry and infection [37], [39], we next examined the interference by heparin with the interaction between CXCR4 and TCA or FS FIV SU. We utilized 3201 cells (CD134^-^, HSPG^-^, feline CXCR4^++^) and SupT1 cells (CD134^-^, HSPG^-^, human CXCR4^++^), which are both strongly bound by FIV SU) [37], as tools to measure CXCR4 binding. Our previous reports [16], [37], [47] showed that: 1) the binding of FIV-SUs to 3201 cells or SupT1 cells is CXCR4 dependent, for both FS and TCA SU; and 2) heparinase I (10 U/ml) treatment has no effect on the inhibition of the binding of 34TF10 SU-Fc to 3201 cells mediated by heparin. Therefore, heparin interferes with an interaction between SU and CXCR4 rather than an interaction between SU and HSPGs in 3201 cells and SupT1 cells. As described above, two TCA (PPRcr and 34TF10) and two FS (PPR and C36) SUs-Fc were studied and an equal amount of each SU was used. We co-incubated SU-Fc adhesins plus heparin (at indicated concentrations) for 45 min, followed by the addition of cells.

**Figure 2:**
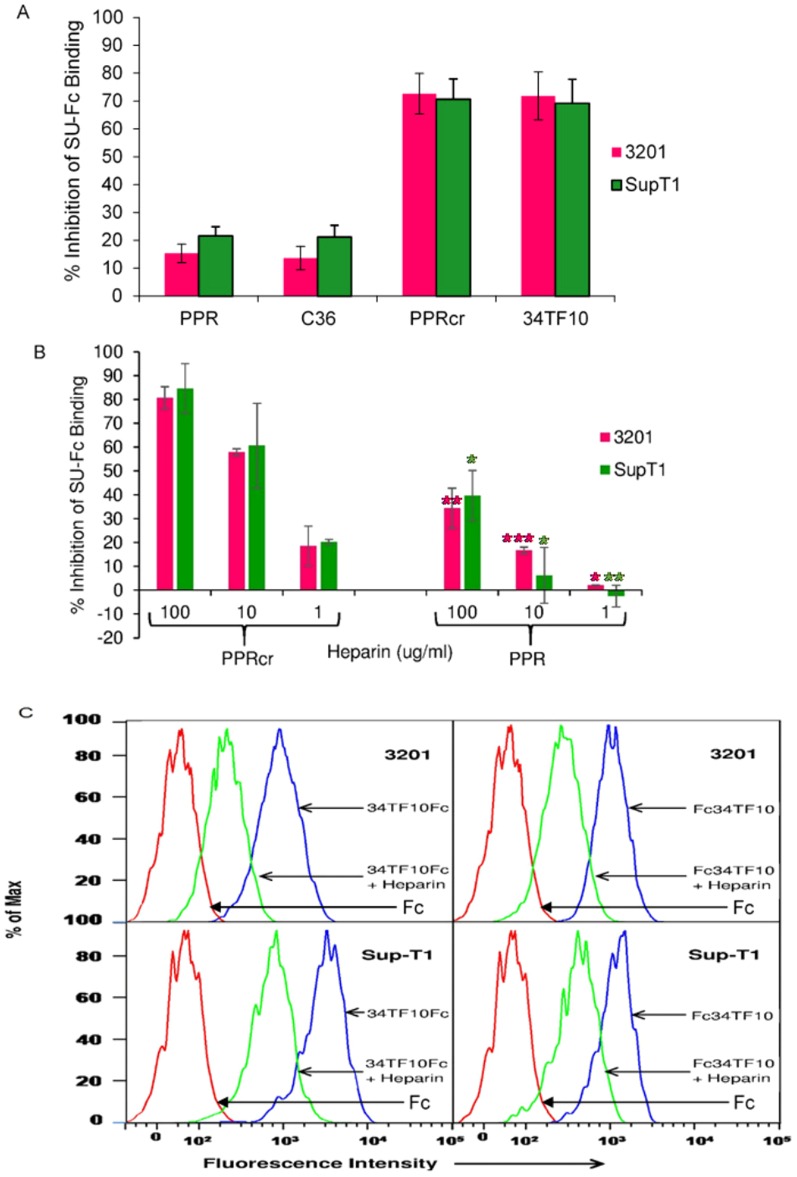
Heparin selectively interferes with TCA SU/CXCR4 interactions. (A) Effect of heparin on TCA or FS SUs-Fc binding to 3201 cells (feline CXCR4) and SupT1 cells (human CXCR4). Heparin was used as 20 µg/ml. Values are inhibition percentage calculated as described in “Materials and Methods”. Results are means and SD for four independent determinations. (B) Heparin interferes with PPRcr or PPR SU-Fc binding to CXCR4 at the indicated concentrations. Values are inhibition percentage. Results are means and SD for three independent determinations. ***p<0.001; **p<0.01; *p<0.05; as compared to the PPR group at the same concentration. (C) Heparin interferes with 34TF10 SU binding to CXCR4. 34 SU-Fc and Fc-34 SU were treated with heparin (20 µg/ml). The top panel and bottom panel represent 3201 cells and SupT1 cells, respectively. Results represent one of three independent experiments.

Our binding competition assay analyzed by FACS (S1 and S2 Figures) indicated that heparin (20 µg/ml) could strongly inhibit PPRcr or 34TF10 SU-Fc binding to 3201 cells, with an inhibition ratio>70% (Fig. 2A); but heparin could not inhibit PPR or C36 SU-Fc binding to the cells, and had a significantly lower inhibition ratio of less than 20%. Statistical analyses (one way ANOVA) using SPSS software indicated that inhibition of FS (PPR and C36) SUs-Fc binding to 3201 cells by heparin was significantly lower than for TCA (PPRcr and 34TF10) SUs-Fc (*p*<0.001). A similar observation (Fig. 2A) was also seen in SupT1 cells, and inhibition of FS (PPR and C36) SUs-Fc binding to SupT1 cells by heparin was significantly lower than those of TCA (PPRcr and 34TF10) SUs-Fc (*p*<0.001). Moreover, when cells were treated with heparin at different concentrations, the results (Fig. 2B) indicated that heparin could inhibit PPRcr SU-Fc binding to 3201 or SupT1 cells in a dose-dependent manner, with an inhibition ratio>80% (100 µg/ml) or around 20% (1 µg/ml). By contrast, when heparin was used at the high concentration of 100 µg/ml, the inhibition ratio for PPR SU-Fc was less than 40% in both cell lines. Statistical analyses indicated that the inhibition ratio of PPR SU-Fc binding to 3201 or SupT1 cells by heparin was significantly lower than that of PPRcr SU-Fc at every concentration (100 µg/ml, 3201 cells----*p*<0.01, SupT1 cells----*p*<0.05; 10 µg/ml, 3201 cells----*p*<0.001, SupT1 cells----*p*<0.05; 1 µg/ml, 3201 cells----*p*<0.05, SupT1 cells----*p*<0.01). Based on these data, we conclude that heparin selectively inhibits PPRcr SU-Fc binding to CXCR4 in a wide range of concentrations of heparin and that heparin selectively inhibits TCA SU binding to CXCR4.

To exclude the possibility that the effect of heparin on SU binding to CXCR4 is dependent on the location of the Fc tag, we compared results using 34SU-Fc and Fc-34SU. Our previous unpublished studies indicated that SU-Fc has much higher binding affinity to CXCR4 than Fc-SU. Thus, the amount of Fc-34SU used was 5 times greater than that of 34SU-Fc in order to obtain similar binding levels. FACS analysis showed that heparin could interfere with both 34SU-Fc (Fig. 2C, left panel) and Fc-34SU (Fig. 2C, right panel) binding to CXCR4 on 3201 cells (Fig. 2C, top panel) or SupT1 (Fig. 2C, bottom panel) cells, confirming that heparin can inhibit 34TF10 SU binding to CXCR4 via the effect on SU itself. We also assessed heparin interference of PPRcr SU constructs (SU-Fc and Fc-SU) for binding to CXCR4; similar results were obtained (data not shown).

Based on the observations described above, we conclude that heparin can effectively inhibit TCA FIV entry and infection, not only due to its strong competition with HSPG, but also to its apparent interference with CXCR4 binding. In contrast, heparin can only moderately inhibit FS FIV entry and temporarily inhibit FS FIV infection at an early stage because of its weak blockade of CD134 and modest hindrance of CXCR4.

### Effect of heparin on PPR mutants binding to CXCR4

**Figure 3:**
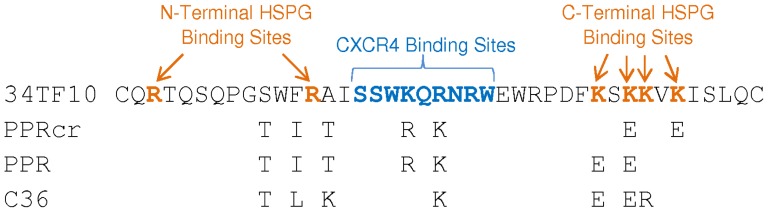
Sequence alignment of the V3 loop of 34TF10, PPRcr, PPR, and C36 surface glycoprotein. Blue indicates the CXCR4 binding region, and orange indicates the HSPG binding region. Both regions are indicated in bold.

It has been reported that heparin or dextran sulfate interacts with HIV-1 through the V3 loop of gp120 [36], [40], and our previous studies [35], [37], [39] also showed that the V3 loop of FIV SU is important for CXCR4 and HSPG interaction (the binding region is shown in Fig. 3). We next investigated whether heparin interfered with FIV TCA SU/CXCR4 via the CXCR4 binding region or HSPG binding region. It has been reported that the CXCR4 binding region (N44 region) is highly conserved between TCA and FS SUs [37], [39], and we can see the N44 region is in fact identical between closely related isolates PPR and PPRcr from Fig. 3. As we know, both FIV TCA and FS SUs bind to CXCR4 through the N44 region [37], [39]. Therefore, if heparin interferes with FIV TCA SU binding to CXCR4 directly through the CXCR4 binding region, the binding of PPR mutants with point mutations in the N44 region to CXCR4 might be inhibited by heparin to an extent similar to PPRcr. To this aim, we prepared a panel of PPR SU-Fc adhesins with mutations in the N44 region to identify changes that might alter the character of FS SU-Fc/CXCR4. When equivalent amounts of mutant PPR SU-Fc and wild type (WT) PPR SU were utilized, all of the mutants maintained around 90% binding ability to CXCR4 compared to WT [37].

**Figure 4:**
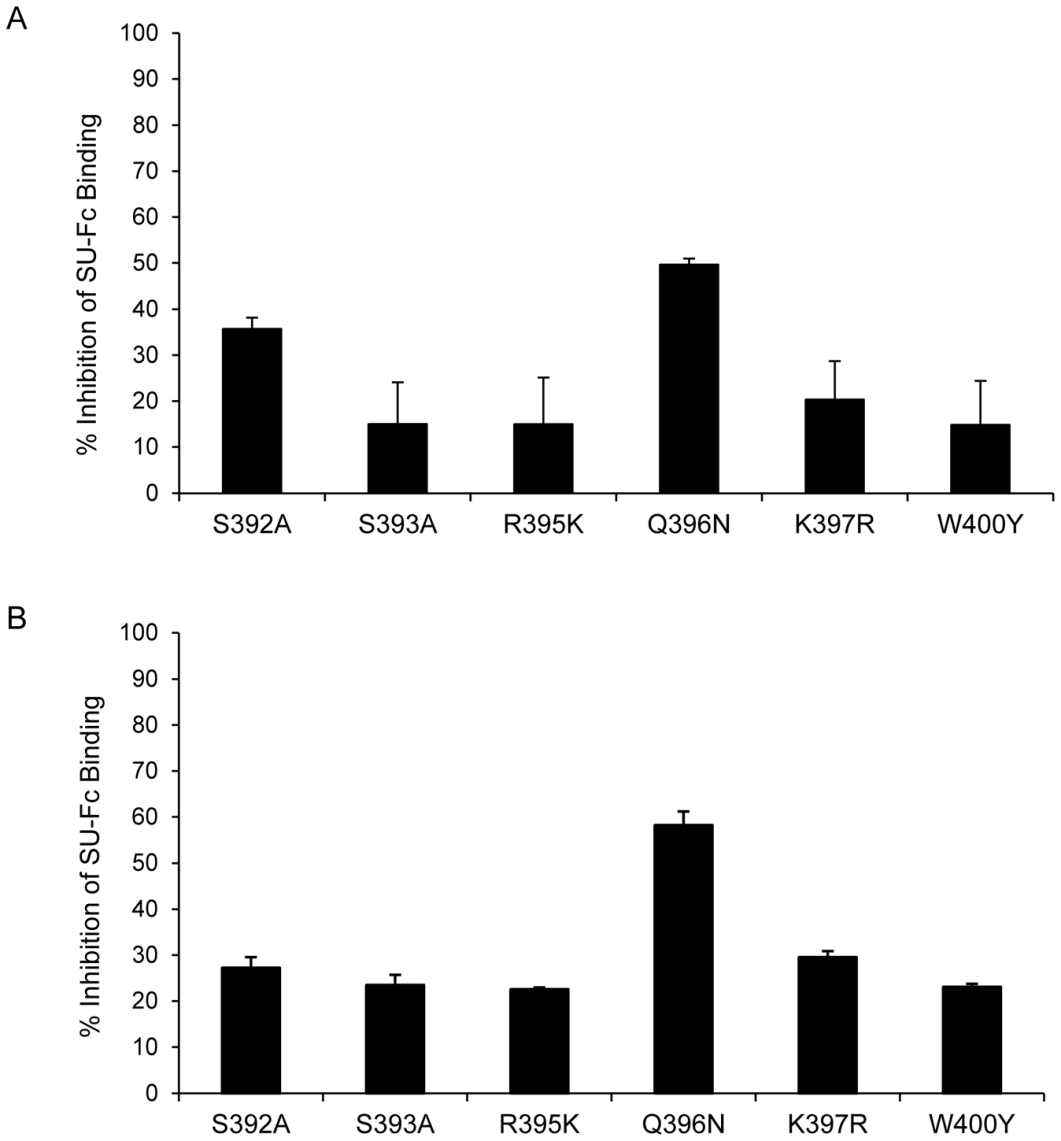
Effect of heparin on PPR mutants binding to CXCR4. Panel A and B represent 3201 cells and SupT1 cells, respectively. Heparin was used at 20 µg/ml. Values are inhibition percentage calculated as described in “Materials and Methods”. Results are means and standard deviations for triplicate determinations.

Results for the inhibition of PPR mutants binding to CXCR4 by heparin are shown in Fig. 4. As can be seen, the inhibition ratios of heparin interference of CXCR4 binding for most mutants was less than 30%, which was similar to the situation with PPR (*p*>0.05) but not PPRcr (*p*<0.01). Although the inhibition ratio of Q396N by heparin was greater than 50%, it was still less than that observed for TCA SUs (*p*<0.05). Thus, our data indicated that the CXCR4 binding region was not directly involved in heparin/SU interactions; however, the extent to which heparin interfered with CXCR4 binding may be influenced by the position or the type of amino acid residue in the N44 region. These results showed that the N44 region of FIV SU was not a target for heparin binding/interference.

### V3 peptides influence heparin effects on TCA FIV SU binding to CXCR4

**Figure 5:**
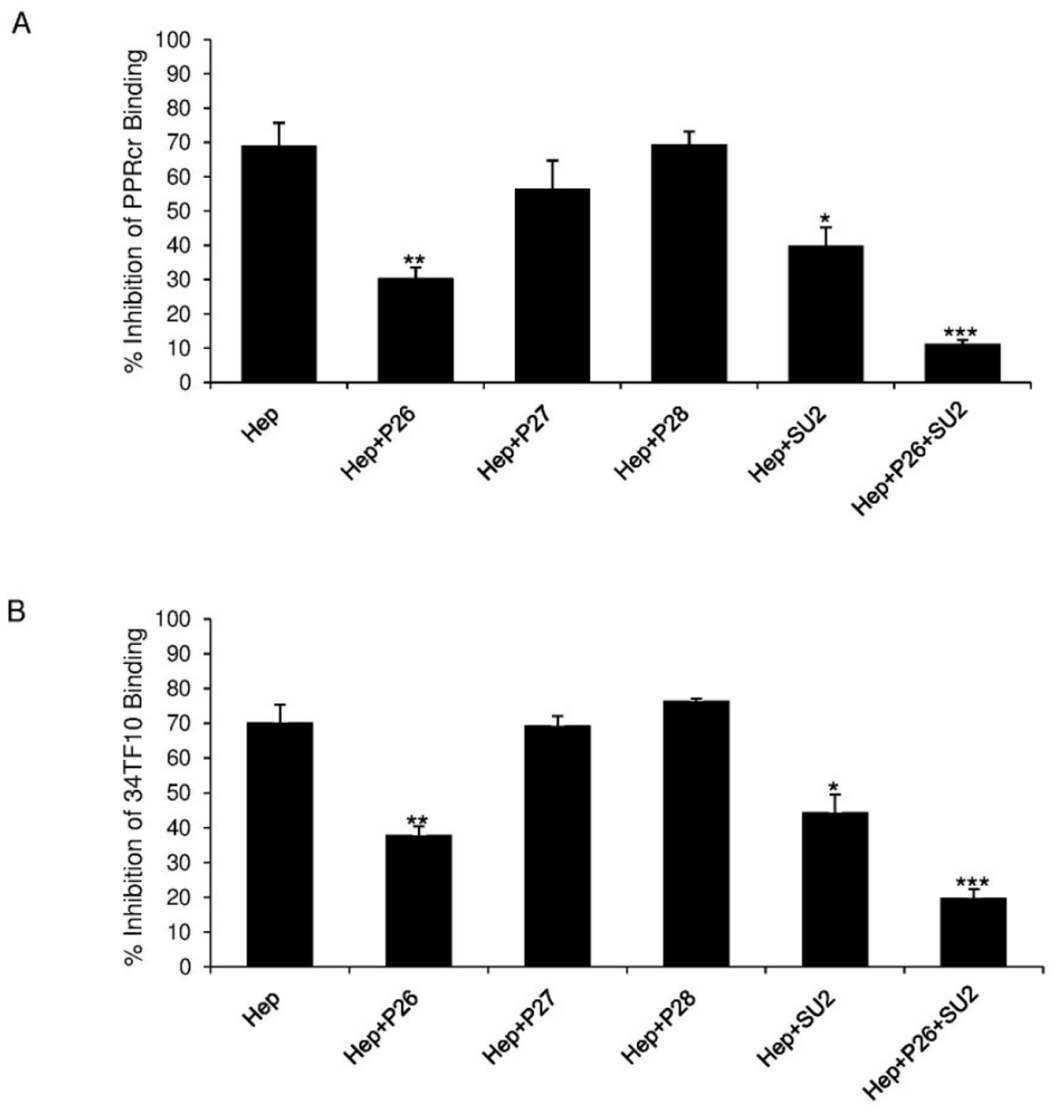
V3 peptide affects heparin interference with TCA FIV SU binding to CXCR4. Panel A and B represent PPRcr and 34TF10 SU-Fc binding to 3201 cells, respectively. All peptides were used at 50 µg/ml as final concentration. Heparin was used at 20 µg/ml. Values are inhibition percentage calculated as described in “Materials and Methods”. Results are means and SD for three independent determinations. ***p<0.001; **p<0.01; *p<0.05; as compared to the heparin treatment group without peptides.

Since heparin is an analogue of HSPG, we next hypothesized that heparin interacts with TCA SU through the HSPG binding region to affect SU/CXCR4 interaction. Previous studies [35] showed that both N-terminal and C-terminal sides of the V3 loop (key amino acid residues are shown on Fig. 3) are critical for the HSPG binding. Based on our previous findings and the character of a set of peptides [35], [39], we chose peptides lacking the CXCR4 binding region but encompassing either the N-terminal or C-terminal portion of V3 to perform the study; peptides P26, P27, P28 and SU-2 were selected, with the sequence shown in Table 1. We next analyzed the influence of these peptides on TCA SU binding to CXCR4 when heparin was co-treated. We pre-incubated heparin with each peptide, then added the mixture plus PPRcr (Fig. 5A) or 34TF10 SU-Fc (Fig. 5B) to 3201 cells. The inhibition of PPRcr or 34TF10 SU-Fc binding to 3201 cells by heparin was used as a control and compared to the inhibition of PPRcr or 34TF10 SU-Fc binding to 3201 cells when co-treated with heparin plus peptide. The results (Fig. 5) showed that P26 (*p*<0.01) and SU-2 (*p*<0.05) could at least partially block the effect of heparin on PPRcr SU-Fc binding to 3201 cells. When P26 and SU-2 were combined, PPRcr SU-Fc binding to CXCR4 inhibited by heparin was almost recovered (*p*<0.001); in contrast, P27 (*p*>0.05) or P28 (*p*>0.05) had no apparent influence on the action of heparin (Fig. 5A). Similar findings were obtained for 34TF10 SU-Fc (Fig. 5B). The data suggested that only V3 peptides containing HSPG binding sites can rescue the SU/CXCR4 interaction inhibited by heparin. Our results here showed that heparin interference of TCA SUs with CXCR4 resulted from its binding to SUs through HSPG binding sites. As we know, peptide P26 and SU-2 can strongly inhibit TCA FIV SU binding to HSPG on the cells [35], which implies that the interaction of V3 peptides with heparin is weaker than with native HSPG, possibly due to secondary structure differences between heparin in solution and HSPG on the cell surface. Based on the data, we hypothesize that TCA FIV SU binding to heparin masks the sites for CXCR4 binding, perhaps as a consequence of conformational changes that occur upon heparin binding.

**Table 1:** Origin and sequence of V3 peptides.

Peptide	Origin	Sequence
P26	PPR	CQRTQSQPGTWIRTISSWRQKN
P27	PPR	TWIRTISSWRQKN
P28	PPR	RWEWRPDFESEKVKISLQC
SU-2	34TF10	QRNRWEWRPDFESEKVKISLQC

Bold and underlined amino acid residues are critical for HSPG binding.

### Heparin affects SU-V3 antibody interactions

It has been reported that heparin and dextran sulfate can block the interactions between HIV-1 envelope and anti-V3 monoclonal antibodies (mAb) [36], [40]. Thus, we analyzed the capacity of heparin to interfere with the interactions between SU and a series of anti-V3 mAbs, to determine whether heparin has distinct influence on the recognition of TCA and FS SUs with anti-V3 mAbs. We utilized a set of anti-V3 mAbs against unique epitopes (Table 2) in the central and C-terminus of the V3 loop and which encompass the CXCR4 binding region or HSPG binding region, respectively.

**Table 2:** Effects of anti-V3 antibodies on PPRcr or 34TF10 SU-Fc binding to HSPG in G355-5 cells.

Antibodies	Recognition Epitopes	Inhibition	
		PPRcr	34TF10
SU1-5	RPDFESEKVK	**–**	**–**
SU1-7	conformation dependent	**+**	**+**
SU1-10	RPDFESEKVK	**–**	**–**
SU1-30	WRQKNRWEWR	**+**	**+**
SU2-4	QKNRWEWRPDF	**+**	**+**
SU2-5	RWEWRPDFES	**+**	**+**
SU2-10	NRWEWRPDF	**+**	**+**

Percent inhibition was calculated as previously described and are expressed as: “+”  = 15–30%; and “–”  = 0–15%. All antibodies (initialed with SU) were used at 50 µg/ml as final concentration. Bold and underline amino acid residue is critical for recognition.

ELISA assays using the anti-V3 mAbs were performed in the presence and absence of heparin. As all seven of the anti-V3 mAbs can recognize PPR SU-Fc, the mean optical density value of PPR SU-Fc is regarded as 100%, and values for binding to other SUs-Fc are percentages of PPR SU-Fc. The results are summarized in Table 3. The recognition regions of antibodies SU1-5 and SU1-10 are located on the C-terminal side of PPR V3 loop and could not recognize 34TF10 or PPRcr SU-Fc, suggesting that the key epitopes recognized by these two antibodies may involve multiple amino acids corresponding to HSPG binding sites, such as E407, E409, K410 and K412 (Fig. 3). FS C36 SU-Fc also could not be recognized by SU1-5. SU1-7, or SU1-10, due to strain specificity for these two antibodies rather than inherent differences between TCA and FS strains. Antibodies SU1-7, SU1-30, SU2-4 and SU2-5 could recognize both PPRcr and 34TF10 SU-Fc to at least 80%, compared with PPR SU-Fc. The affinity of SU2-10 for 34TF10 SU-Fc binding was much lower, binding to only appr. 40% the level of PPR SU-Fc binding, while PPRcr and C36 SU-Fc bound 60–80% the level of PPR SU-Fc. SU1-30, SU2-4 and SU2-5 mAbs could bind C36 SU-Fc greater than 60% compared with PPR SU-Fc. The differences of SUs-Fc affinities observed within the various anti-V3 mAbs indicate that one specific anti-V3 mAb may bind to the same V3 epitopes in different conformations on SU-Fc. Overall, there were no major differences for SU-Fc binding to anti-V3 mAbs in the presence or absence of heparin, no matter whether the isolate was a TCA or FS SU, suggesting that heparin does not mask epitopes on SU recognized by anti-V3 mAbs.

**Table 3:** Determination of binding of SU/anti-V3 antibodies by ELISA, in the presence or absence of heparin.

Antibodies	PPR		PPRcr		C36		34TF10	
	-	+	-	+	-	+	-	+
SU1-5	100.0	108.4	**2.4**	**−0.4**	**5.9**	**6.9**	**1.3**	**−4.4**
SU1-7	100.0	98.9	91.0	85.7	34.7	33.9	80.9	67.8
SU1-10	100.0	94.4	**1.2**	**0.0**	**5.7**	**6.4**	**2.2**	**−0.5**
SU1-30	100.0	98.0	94.1	79.7	65.2	66.6	80.5	68.7
SU2-4	100.0	100.5	96.1	90.8	88.0	84.0	94.6	83.2
SU2-5	100.0	96.9	96.9	85.6	84.7	83.2	92.4	82.1
SU2-10	100.0	100.1	76.9	64.7	64.9	57.5	41.6	31.6

Binding of FIV SU-Fc to anti-V3 antibodies detected by ELISA as described in “Materials and Methods”. Anti-V3 mAbs were incubated with SU-Fc for 1 h in the presence or absence of heparin at room temperature. Values for other SUs-Fc are percentages of the mean optical density value of PPR SU-Fc, which is regarded as 100%. Results represent one of three independent experiments. Values in bold represent loss of specific antibody reactivity to a given SU-Fc.

To determine whether the inability of heparin to interfere with the interaction of SU-V3 antibodies was because antibody-sensitive epitopes have no overlapping HSPG binding sites, we next evaluated the effects of antibodies on HSPG binding in G355-5 cells. The results (summarized in Table 2) indicated that two antibodies, SU1-5 and SU1-10, which could not recognize PPRcr and 34TF10 SUs, had no inhibition in HSPG binding. For the other five mAbs, they had mild effects on interfering with binding of PPRcr and 34TF10 SUs-Fc to HSPG. As expected, none of these anti-V3 mAbs interfered with SU/HSPG interaction, which explained the lack of influence of heparin on SU/anti-V3 antibody interactions described above and implied that none of these antibodies target the HSPG region. The outcomes further confirmed that five of these anti-V3 mAbs, except SU1-5 and SU1-7, block SU/CXCR4 interaction and antibody-sensitive epitopes may be located in the CXCR4 binding region or in very close proximity to this domain [37], [39].

To confirm the neutralization capacities of anti-V3 mAbs on TCA FIV, we measured the neutralization effects on 34TF10 entering G355-5 cells. The results (S3 Figure) showed that most of the anti-V3 antibodies had strong neutralization effects on 34TF10 entering G355-5 cells, with IC_50_ approximately 0.1–2.5 µg/ml; the exceptions were SU1-5 and SU1-10, both with IC_50_>200 µg/ml. Thus, the weak neutralization effect on 34TF10 pseudotyped virions entering G355-5 cells (S3 Figure) and the lack of influence on HSPG binding of PPRcr or 34TF10 SU-Fc to G355-5 cells (Table 2) by SU1-5 and SU1-10 were due to the inability of these antibodies to bind TCA FIV. Compared with other sensitive mAbs such SU1-7, SU1-30, SU2-4 and SU2-5, the neutralization effect of SU2-10 was relatively weak, which was also consistent with poor recognition capacity. The observation that sensitive mAbs neutralized infection to different extents is consistent with slight variations in neutralization epitopes within V3.

## Discussion

Feline immunodeficiency virus (FIV) is the only non-primate lentivirus that causes an AIDS-like disease in the domestic cat [41]. FIV infection in cats therefore has been established as a valuable animal model for the development of anti-HIV therapy [42], [43]. HSPG play an important role in the infection of cells by HIV-1 [44], [45] and the interaction of HSPG with HIV V3 loop is a potential target for anti-HIV therapy by preventing HIV from entering cells [19]. Similarly, laboratory-adapted strains (TCA) of FIV can bind to HSPG through the HSPG binding region around the V3 loop and thus productively infect adherent cell lines such as CrFK and G355-5 [35]. It will be of great significance to elucidate the molecular mechanism(s) of action for heparin on FIV infection for the development of anti-HIV therapeutics.

Our study indicated that heparin can selectively inhibit the productive infection of FIV TCA, but not FS FIV (Fig. 1), which means heparin selectively interacts with TCA, consistent with the report that polyanions (such as heparin and dextran sulfate) selectively inhibit HIV infection of X4 or R5X4 but not R5 [36].

Our binding competition assays showed that heparin can strongly inhibit TCA SU-Fc binding to CXCR4, with an inhibition ratio>70%, but cannot inhibit FS SU-Fc binding to CXCR4 (inhibition ratio <30%; Fig. 2). Furthermore, peptide P26 (encompassing the N-terminal portion of V3) combined with SU-2 (encompassing the C-terminus) could almost recover the interference with TCA FIV SU binding to CXCR4 by heparin, suggesting that heparin binds to TCA SU via HSPG binding region within the V3 loop, which may cover-up the sites for CXCR4 binding or lead to CXCR4 binding conformation change. The negative effect of heparin on the interaction of SU/V3 antibody is expected (Table 3), given that none of the anti-V3 mAbs directly target the HSPG binding motifs, although SU1-5 and SU1-10, which contain the HSPG binding domain but not the correct types of amino acids for binding, while the other five antibodies target the CXCR4-binding region (Table 2). In addition, one explanation is that our non-sulfated antibodies contain no analogous sulfated motifs that permit SU contact and bind well to TCA SU, since it has been reported that non-sulfated anti-gp120 antibodies block HIV-1 binding to syndecan (one kind of HSPG) less efficiently than the sulfated ones [46].

Based on the data, we can conclude that heparin blocks TCA FIV infection or entry not only by competition of HSPG on the cell surface interaction with SU, but also by interference with CXCR4 binding to SU. Since certain strains of both FIV and HIV use HSPG and CXCR4 for infection, it is attractive to design and synthesize heparin-derivatives or analogues that can work on both steps for HSPG and CXCR4 interaction with SU for HIV treatment. Due to the negative effect of heparin on the interaction of SU with anti-V3 antibodies targeting CXCR4-binding region, it is possible to enhance the inhibitory effect for FIV treatment by combination of heparin with anti-V3 antibodies targeting CXCR4 binding region. We are performing the studies to test the hypothesis. Our studies offer useful information to improve our understanding of the precise interactions between the viral envelope and cell entry receptors.

It appears that the use of heparin analogues as anti-HIV therapeutic agents is not promising in vivo. One reason for the failure in clinics may be their weak neutralization activity for R5 viruses, the viral phenotype associated with HIV-1 transmission and early infection [36]. Another reason is that HIV resistant variants are rapidly generated [36]. However, several routes [57] can generate heparin derivatives with low anticoagulant and high anti-HIV-1 activities in vitro, which are good candidates for clinical investigation as potential novel therapeutic agents combined with other drugs to control AIDS and HIV infection [57]. And a series of chemically modified heparins have shown that there is structural specificity in the anti-HIV activity [57]. Therefore, our findings together with other previous studies [57] give the chance to rationally develop small-molecule inhibitors based on electrostatic interactions with envelope protein.

Sequences have been determined for relatively few HS chains of HSPG, and there have been relatively few studies of HS–protein binding at the molecular level, mainly due to the relatively small amounts of HS and HS-derived oligosaccharides that can normally be obtained from cell surfaces. In contrast, more abundant heparin and heparin-derived oligosaccharides have been used as models for HS [19]. Therefore, studies on heparin/SU will not only be of use in designation or modification heparin derivatives for the treatment of FIV or HIV infections, but also supplement the knowledge for HSPG/SU interaction.

## Materials and Methods

### Cell lines, virus and reagents

The feline glial cell line (G355-5) was kindly provided by Don Blair (National Institutes of Health, Bethesda, MD), with high heparin sulfate proteoglycans (HSPG) expression; low CXCR4 expression, and negative for CD134 expression [16], [47]. Gfox cells are Crandell feline kidney (CrFK) cells stably transfected with feline CD134 and thus productively infectable by field strains of FIV [48], [49]. The IL-2-independent feline lymphoma cell line 3201 was obtained from William Hardy (Sloan-Kettering Memorial Hospital, NY). 3201 cells have high CXCR4 expression, with a very low or negative expression of CD134 and HSPG [47]. Human T-lymphocytic SupT1 cells expressing CXCR4 were acquired from Bruce Torbett [58] and cultivated in DMEM medium supplemented with 10% fetal bovine serum (FBS, Invitrogen, Carlsbad, CA), 2 mM L-glutamine (Sigma, St. Louis, MO), non-essential amino acids (Sigma), Penicillin-Streptomycin 100 u/ml and 100 µg/ml respectively (Invitrogen). Propagation of the different cell lines was performed as previously described [16], [37], [39], [50]. The FIV strains used in the present study described as below: FIV-PPR, is a molecular clone of the clade A San Diego isolate [51]. PPR_CrFK_ (PPRcr) is the virus outgrowth from continuous passage of FIV-PPR on CrFK cells for a period of approximately three weeks [35], namely, a FIV-PPR strain obtained after *ex vivo* passage in the CrFK cell line [35]; PPRcr can bind to HSPG-expressing adherent cell lines (such as G355-5 or CrFK cell line) and productively infect those cell lines. At the same time, PPRcr maintains the ability to infect CrFK cells engineered to over-express CD134 (Gfox cells) [35]. FIV-34TF10 is a molecular clone of the FIV Petaluma isolate that had been adapted for growth on CrFK cells [52]. FIV-C36 is a highly pathogenic molecular isolate of feline immunodeficiency virus subtype C [53]. Fetal bovine serum was obtained from Invitrogen (Calsbad, CA). Heparin and bovine serum albumin (BSA) were purchased from Sigma (St. Louis, MO).

### Virus infection assay

Viruses with RT values above 100K CPM were used in all infection assays. Equivalent infectious doses of each virus were incubated with 20 µg/ml heparin (diluted in cell media) for 30 minutes at room temperature. Then, 2×10^4^ cells (G355-5 or Gfox) were seeded in a 12-well plate with the virus/heparin mixtures and were spinoculated for 2 hrs at 3,000 rpm. The samples were then placed in the incubator to recover for 2 hrs before the samples were washed once by doing a quick spin, dumping the media, and replacing with fresh media containing fresh heparin. Samples were cultured at 37°C in a 5% CO_2_ atmosphere. Every 3 days, medium was replaced by fresh heparin diluted in cell media. Virus production was measured over time using a micro-RT assay. To compare the CPM values before and after treatment, an independent sample t-test was employed. ***p<0.001; **p<0.01; *p<0.05.

### Micro-RT activity assay

Micro-RT activity assay was performed as previously described [38] Briefly, 50 µl of cell-free supernatant together with 10 µl of lysis buffer (0.75 M KCl, 20 mM dithiothreitol, 0.5% Triton X−100) was incubated at room temperature for 10 minutes. Then, 40 µl of a mixture containing 125 mM Tris-HCl (pH 8.1), 12.5 mM MgCl_2_, 1.25 µg poly(rA)-poly(dT)_12–18_ (Amersham Biosciences, Piscataway, NJ) and 1.25 µCi of [^3^H]dTTP (DuPont, Boston, MA) was added to the sample followed by 2 h of incubation at 37°C. The measurement of RT activity was previously described [38].

### Virus entry assay

pCFIV hybrid vectors pseudotyped with FIV-34TF10, PPRcr, PPR, or C36 envelope genes were co-transfected with a beta-galactosidase (β-gal)-expressing packaging vector in 293T cells [56]. Two days later, viral supernatants were collected before performing a single round infection assay in G355-5 or Gfox cells in the presence or absence of heparin at indicated concentrations. After 48 hours of infection, β-gal activity was measured with the Tropix Galacto-Star chemiluminescent reporter gene assay (Applied Biosystems, Carlsbad, CA) according to the manufacturer's guidelines. For neutralization studies, all antibodies were pre-incubated with pseudovirions at 37°C for 60 min at indicated concentrations, and then co-treated with G355-5 cells to be assayed for betagalactosidase expression as described above. Percent inhibition was calculated by the formula 100−[(*t−c*)/(*m−c*) ×100], where *t* represents the signal for heparin or antibodies treatment; *c* represents the background signal in the absence of pseudovirions; and *m* represents the signal obtained for pseudovirions in the absence of heparin or antibodies. To compare inhibition ratio among groups, One-factor Analysis of Variance (one way ANOVA) using SPSS software was employed. ***p<0.001; **p<0.01; *p<0.05.

### Recombinant SU proteins

Expression plasmids encoding SU of FIV-PPR, FIV-PPRcr, FIV-C36, FIV-34TF10 and PPR with point mutations were constructed and used for production of stable CHO-K1 cell lines, as previously described [16], [35], [47], [54], [55]. Single colonies with high expression of desired Fc-tagged proteins were selected and SU-Fc or Fc-SU fusion proteins (adhesins) were purified as described before [37] and proteins with purity>95% were used. The adhesins were quantified by a human IgG ELISA quantitation kit (Bethyl Laboratories, Inc, Montgomery, TX), and the same amount of adhesins were employed in the following binding assay and ELISA assay. Finally, relative quantitation of proteins was confirmed or adjusted by western blot analysis, as previously described [37].

### Flow cytometry analysis

Binding of SU-Fc or Fc-SU adhesins or Fc (negative control) to the surfaces of 3201, SupT1 or G355-5 cells were detected using a phycoerythrin-conjugated goat anti-human IgG1 Fc antibody (MP Biomedicals, Aurora, OH) and analyzed by flow cytometry, using FLOWJO software (Tree Star, San Carlos, CA). Briefly, for binding to 3201 or SupT1 cells, 100 ng of SU-Fc adhesins or Fc was co-incubated with heparin at indicated concentrations for 45 min, followed by the addition of 1.5×10^5^ cells and incubated for another 45 min at 25°C. The procedure was performed in EBSS–2% FBS buffer. After washing, cells were labeled with a 1∶1000 dilution of PE-conjugated goat anti-human IgG1 antibody (MP Biomedicals, Aurora, OH) for 35 min at 25°C. SU-Fc binding was monitored by FACS analysis. For V3 peptide competitive binding studies, we pre-incubated heparin with each peptide for 45 min at 25°C, then added the mixture plus SU-Fc to 3201 cells. The following process was the same as described above. For the binding to G355-5 cells, PPRcr or 34TF10 SU-Fc (500 ng) was pre-incubated with the various anti-V3 mAbs for 30 min before the addition to 1×10^5^ G355-5 cells. Then the cells in EBSS–0.1% bovine serum albumin were incubated at 4°C for 45 min. Finishing process was similar as above. Percent inhibition was calculated by the formula 100−[(*t−c*)/(*m−c*) ×100], where *t* represents the signal for the test sample; *c* represents the background signal in the presence of Fc control; and *m* represents the signal obtained for SU-Fc in the absence of heparin, antibodies, or peptides. To compare inhibition ratio among groups, One-factor Analysis of Variance (one way ANOVA) using SPSS software was employed. If only compare inhibition ratio between two groups, an independent sample t-test was employed. ***p<0.001; **p<0.01; *p<0.05.

### Enzyme-linked immunosorbent assay (ELISA)

Immulon II HB plates (Thermo, Milford, MA) were coated overnight with 1 µg of anti-V3 antibodies in PBS (pH 7.2). Then the plate was washed twice with PBS and dried. The same amount of purified SU-Fc fusion proteins was diluted in 100 µl of ELISA buffer (0.15 M NaCl, 0.05 M Tris-HCl, 1 mM EDTA, 3% bovine serum albumin fraction V, 3.5% fetal calf serum, and 0.05% Tween 20, pH 7.4) in the presence or absence of heparin, then added to appropriate wells. After 1.5 h incubation, the plate was washed three times with PBS and dried. HRP- conjugated goat anti-human IgG antibody diluted in ELISA buffer was added to every well and incubated for 1 h. After the same washing procedure, enzyme substrate reaction was performed for 10 min by using OPD as substrate, followed by the addition of stop solution 2 M H_2_SO4. The OD value was read at 493 nm using a microtiter plate reader. All operations were carried out at room temperature. For measurement of the binding of antibodies to SU-Fc, various dilutions of SU-Fc were performed in a pilot experiment to ensure that binding occurred in the linear range of the assay.

## Supporting Information

S1 Figure **Interference with FIV SU/CXCR4 interactions by heparin.** (A). Effect of heparin on FIV PPR, C36, PPRcr, and 34TF10 SUs-Fc binding to 3201 cells (feline CXCR4). (B). Effect of heparin on FIV PPR, C36, PPRcr, and 34TF10 SUs-Fc binding to SupT1 cells (human CXCR4). FACS analysis was performed by using the same amount of SUs-Fc. Fc was utilized as a control. Heparin was used at 20 µg/ml. Results are representative of four independent determinations.
(TIF)

S2 Figure **Heparin interferes with PPRcr or PPR SU-Fc binding to CXCR4 at the indicated concentrations.** Left and right panel shows the effect of heparin on FIV PPR and PPRcr SUs-Fc binding to 3201 and SupT1 cells, respectively. FACS analysis was performed by using the same amount of SUs-Fc. Fc was utilized as a control. Heparin was used at the indicated concentrations. Results are representative of three independent determinations.
(TIF)

S3 Figure **Neutralization Effects of anti-V3 antibodies on 34TF10 entry into G355-5 Cells.** All antibodies were pre-incubated with pseudovirions at 37°C for 60 min at indicated concentrations, and then co-treated with G355-5 cells to perform entry assay. β-gal assays were analyzed 48 h after infections. Values are inhibition percentage calculated as described in “Materials and Methods”. Results are means and standard deviations (SD) for three independent determinations.
(TIF)
