# Supplementary figures and images for: Selective Interaction of Heparin with the Variable Region 3 within Surface Glycoprotein of Laboratory-Adapted Feline Immunodeficiency Virus
Source: PLoS One. 2014 Dec 18;9(12):e115252. doi: 10.1371/journal.pone.0115252 (PMC4270745; doi:10.1371/journal.pone.0115252)

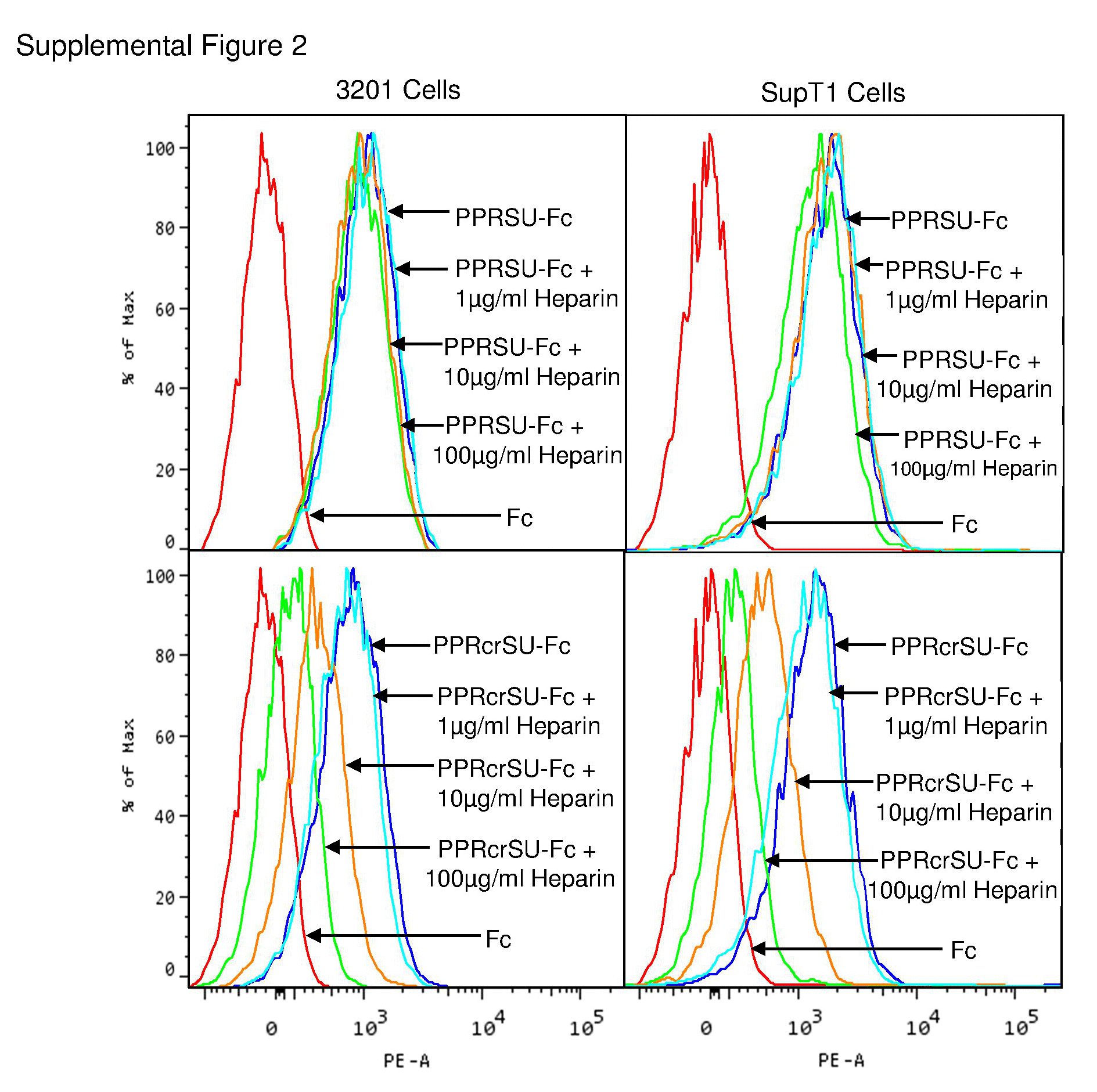

Supplement: S2 Figure — Heparin interferes with PPRcr or PPR SU-Fc binding to CXCR4 at the indicated concentrations. Left and right panel shows the effect of heparin on FIV PPR and PPRcr SUs-Fc binding to 3201 and SupT1 cells, respectively. FACS analysis was performed by using the same amount of SUs-Fc. Fc was utilized as a control. Heparin was used at the indicated concentrations. Results are representative of three independent determinations. (TIF) [file pone.0115252.s002.tif]
